# Evaluation of a Sepsis Alert System at a Veterans Affairs Medical Center

**DOI:** 10.1017/ash.2024.178

**Published:** 2024-09-16

**Authors:** Grace Roberts, Bushra Akbar, Jessica Bennett, Anna Mitchell, Neena Thomas-Gosain

**Affiliations:** University of Tennessee Health Science Center; Lt. Col Weathers Jr VA Medical Center; Department of Veterans Affairs; University of Colorado

## Abstract

**Background:** Automated sepsis alerts have become a widely implemented screening tool aimed at early detection of clinically unstable patients. Prior research has shown mixed results depending on the type of screening tools used and the patient population studied. This study aimed to evaluate the predictive value of an alert system created for identifying patients with sepsis to determine utility in clinical practice prior to implementation. Additionally, clinical management of those with and without sepsis was compared to measure potential added benefit of this system in clinical decision making. **Methods:** A TheraDoc® software sepsis alert was generated for non-ICU patients meeting >2 SIRS criteria within a 24-hour time period (temperature >38°C or 90, respiratory rate >20 or partial pressure CO2 12,000 or 10% bands/immature cells) during March 2023. Alerts were excluded if they were duplicates (using identical criteria or a second alert within 24 hours), triggered by labs collected >48 hours prior, or death or discharge occurred before the time of alert. The primary outcome was positive predictive value (PPV) of sepsis identification, confirmed by ICD-10 codes and diagnostic studies (cultures, imaging). Secondary outcomes included clinical management (antibiotic utilization [AU] and choice, infectious disease [ID] consultations and culture collection). Antibiotics were categorized as broad-spectrum using National Healthcare Safety Network (NSHN) criteria. Secondary outcomes were compared between sepsis and SIRS without infection groups (SIRS) by chi-square analysis. **Results:** After applying exclusion criteria, 116 of 166 alerts were analyzed; 55 of 116 alerts had confirmed sepsis (PPV 47.4%). Patients with sepsis were more likely to have an ID consult (16% [9/55] vs 7% [4/61]) and cultures collected (70.9% [39/55] vs 39.3% [24/61]) compared to SIRS patients, however these differences were not statistically significant. AU was higher with confirmed infections compared to SIRS patients (94.5% [52/55] vs 32.8% [20/61], p < 0 .05) along with use of broad-spectrum antibiotics (73% [38/52] vs 40% [ 8/20] p < 0 .05). **Conclusions:** While automated alerts may enable early identification of sepsis, use of SIRS criteria alone has poor specificity, which was borne out by the low PPV in this study. Our study found that management of sepsis patients (as measured by AU and culture ordering) was better than expected and combined with the low PPV of this alert system resulted in our team rejecting widespread adoption of SIRS-based sepsis alerts.

